# Quantification of dose differences between two versions of Acuros XB algorithm compared to Monte Carlo simulations — the effect on clinical patient treatment planning

**DOI:** 10.1120/jacmp.v16i6.5642

**Published:** 2015-11-08

**Authors:** Jarkko Ojala, Mika Kapanen

**Affiliations:** ^1^ Department of Oncology Unit of Radiotherapy, Tampere University Hospital Tampere Finland; ^2^ Department of Medical Physics Medical Imaging Center, Tampere University Hospital Tampere Finland

**Keywords:** dose calculation algorithm, Acuros XB, AXB, Monte Carlo dose calculation, photon beam radiotherapy

## Abstract

A commercialized implementation of linear Boltzmann transport equation solver, the Acuros XB algorithm (AXB), represents a class of most advanced type ‘c’ photon radiotherapy dose calculation algorithms. The purpose of the study was to quantify the effects of the modifications implemented in the more recent version 11 of the AXB (AXB11) compared to the first commercial implementation, version 10 of the AXB (AXB10), in various anatomical regions in clinical treatment planning. Both versions of the AXB were part of Varian's Eclipse clinical treatment planning system and treatment plans for 10 patients were created using intensity‐modulated radiotherapy (IMRT) and volumetric‐modulated arc radiotherapy (VMAT). The plans were first created with the AXB10 and then recalculated with the AXB11 and full Monte Carlo (MC) simulations. Considering the full MC simulations as reference, a DVH analysis for gross tumor and planning target volumes (GTV and PTV) and organs at risk was performed, and also 3D gamma agreement index (GAI) values within a 15% isodose region and for the PTV were determined. Although differences up to 12% in DVH analysis were seen between the MC simulations and the AXB, based on the results of this study no general conclusion can be drawn that the modifications made in the AXB11 compared to the AXB10 would imply that the dose calculation accuracy of the AXB10 would be inferior to the AXB11 in the clinical patient treatment planning. The only clear improvement with the AXB11 over the AXB10 is the dose calculation accuracy in air cavities. In general, no large deviations are present in the DVH analysis results between the two versions of the algorithm, and the results of 3D gamma analysis do not favor one or the other. Thus it may be concluded that the results of the comprehensive studies assessing the accuracy of the AXB10 may be extended to the AXB11.

PACS numbers: 87.55.‐x, 87.55.D‐, 87.55.K‐, 87.55.kd, 87.55.Qr

## I. INTRODUCTION

In radiotherapy, the accuracy of dose calculation plays an important role in the total uncertainty of the whole treatment process. In 2004, the AAPM TG65 estimated that the uncertainty related to dose calculation ranged between 1.0% and 5.0%, but will decrease below 3.0% in the future.^(1)^ Regardless, the relative contribution of the dose calculation uncertainty seems to increase, since the other sources of the total uncertainty were estimated to decrease. The dose calculation algorithms implemented in clinical treatment planning systems (TPS) can be divided in three classes. Knöös et al.^(2)^ proposed the classification for type ‘a’ and type ‘b’ algorithms in 2006 and, in Ojala et al.,^(3)^ the group of type ‘c’ algorithms was introduced. Type ‘a’, correction‐based algorithms, based on measurements and corrected to account for patient contours and tissue heterogeneities, are replaced by type ‘b’, model‐based algorithms. Type ‘b’ algorithms apply various superposition and convolution techniques to provide increased accuracy over type ‘a’ algorithms, especially in the presence of tissue heterogeneities. However, type ‘b’ algorithms have been shown to produce clinically unacceptable discrepancies in the extreme ends of the density range in biological tissues (e.g., air, lung, and bone) and in high‐Z implanted materials (here, high‐Z materials are regarded as materials with larger mass density than found in human tissues). Type ‘c’ algorithms represent the most recent class of dose calculation algorithms which 1) have improved modeling of secondary electron transport essential for accurate heterogeneity correction, when compared to type ‘b’ algorithms; 2) are able to calculate the dose deposition, in addition to all biological tissues, also in the presence of high‐Z implanted materials; and 3) are able to report the dose as dose to medium.^4^, ^5^

The first candidate for the group of type ‘c’ algorithms and the only commercial grid‐based linear Boltzmann transport equation (LBTE) solver for radiotherapy purposes is the Acuros XB (AXB) algorithm implemented in the Eclipse TPS by Varian Medical Systems, Inc. (VMS) (Palo Alto, CA).^(6)^ The foundations of the AXB algorithm are in the general‐purpose radiation transport Attila LBTE solver and in the original Acuros LBTE solver designed for radiotherapy dose calculations, both originating from Transpire, Inc. (Gig Harbor, WA).^7^, ^8^, ^9^, ^10^, ^11^ The first implementation of the AXB algorithm was released in Eclipse TPS version 10.0 (AXB10). Since then, for the version 11.0 update such modifications were included that have reported effects on the dose calculation accuracy.^12^, ^13^, ^14^ As reported in Ojala,^(5)^ the majority of the studies, where the AXB algorithm has been benchmarked and validated for clinical use, in homogeneous, heterogeneous, anthropometric, anthropomorphic phantoms and with patient plans, have applied the AXB10, which means that the effects of modifications implemented in the AXB11 remain largely unreported. In a study by Fogliata et al.,^(12)^ the performance of the AXB10 and the AXB11 was assessed against measurements in a homogeneous water phantom. For open fields, the accuracy of both versions was similar — within 1%. But for fields with mechanical wedges, the AXB11 was reported to produce more accurate results. In another study by Fogliata et al.,^(13)^ the AXB10 and the AXB11 were compared against the analytic anisotropic algorithm (AAA) by VMS and a ‘fast’ Monte Carlo‐based algorithm (the Voxel Monte Carlo – VMC++) in heterogeneous phantoms, including materials representing air and lung, adipose, cartilage and bone tissues. The AXB11 was reported to produce better agreement than the AXB10 with the VMC++ algorithm near heterogeneity interfaces and in tissues of very low densities. Finally, in another study by Fogliata et al.,^(14)^ the AXB10, the AXB11, and the AAA were compared in case of breast radiotherapy. The paper represents the only study where the differences between the AXB10 and the AXB11 are quantified in a clinical setting. Although the reported deviations between the algorithms for parameters considered in clinical practice were rather small, the discrepancies found in more detailed substructural analysis were interpreted as an improvement in accuracy, favoring the dose distributions of the AXB algorithm, especially the ones of the AXB11 in the regions of low‐density lung.

In this study, the aim is to quantify the effects of the modifications implemented in the AXB11 compared to the AXB10 in various anatomical regions. The patient cases are selected to cover various regions of the body, including regions of the extreme ends of the density range (i.e., from air cavities to high‐Z implanted materials). Also, the chosen patient plans are selected so that various treatment techniques (intensity‐modulated radiotherapy (IMRT) and volumetric‐modulated arc radiotherapy (VMAT)) and various fractionations (from hypofractionated stereotactic (body) radiotherapy (SRT/SBRT) treatments to conventional 2 Gy‐per‐day fractionation schemes) are included. The treatment plans are first calculated with the AXB10, then recalculated with the AXB11 and absolute dose calibrated full MC model, which is the reference method. Detailed comparison is performed between the AXB10/AXB11 and the full MC simulations, which, when appropriately commissioned and validated, are considered the most accurate methods to produce dose distributions in 3D heterogeneous complex calculation geometries, such as patient anatomy.^(15)^ With the results of this study, the results obtained with one version of the AXB algorithm can be transformed for those of other versions, and the clinical impact of the differences between the algorithm versions in various anatomical regions can be assessed.

## II. MATERIALS AND METHODS

### A. The AXB algorithm

In this study, version 11.0 of the Eclipse TPS and versions 10.0.28 and 11.0.31 of the AXB algorithm were used. The algorithms were configured for the Varian Clinac iX (2300C/D) linear accelerator (linac) equipped with Millennium 120 MLC. The configuration was performed strictly following manufacturer's manuals and its recommendations and selecting default configuration settings. The effective target spot size was 1.0 mm in both X and Y directions. The MLC leaf transmission was 1.4% and dosimetric leaf gap was 0.19 cm. The measurements were performed with IBA unshielded stereotactic semiconductor field detector (model DEB050; IBA Dosimetry AB, Uppsala, Sweden) for $2\times 2\;\text{to}\;4\times 4\;{\text{cm}}^{2}$ fields and with PTW TM31002 Semiflex $0.125\;{\text{cm}}^{3}$ ionization chamber (IC) (PTW Freiburg GmbH, Germany) for $3\times 3\;\text{to}\;40\times 40\;{\text{cm}}^{2}$ fields using a motorized scanning system in an MP3 water phantom (PTW Freiburg GmbH). The smallest field size in output factor configuration was $2\times 2\;{\text{cm}}^{2}$ and the measurement results were "daisy chained", as presented by Dieterich and Sherouse.^(16)^

The dose calculation by the AXB algorithm can be divided in two stages. In the first stage the radiation beam propagation in the linac treatment head is simulated. The AXB algorithm applies the same subsource models as implemented in the AAA. That is to say that the model contains subsources for: 1) primary photons, which are generated in the X‐ray target but not interacted elsewhere in the treatment head; 2) extrafocal photons, which are generated in interactions in other treatment head components; and 3) electron contamination, which represents the electrons generated in the treatment head components and in the air.^17^, ^18^, ^19^, ^20^, ^21^, ^22^ The source model determines the radiation beam fluence, which is modulated by the plan‐specific jaw and MLC configurations, and is then delivered to the patient. In the patient dose calculation, the following steps are performed:

transport of the source model fluence into the patient;
2. calculation of scattered photon fluence in the patient;
3. calculation of scattered electron fluence in the patient, and
4. dose calculation.

Step 1 is repeated for each field direction, and steps 2 to 3 are performed only once for each patient geometry voxel. In the final step 4, the absolute dose in each voxel is calculated using the determined electron angular fluence, macroscopic electron energy deposition cross sections, and material density of the voxel.^6^, ^11^ The material mapping needs to be done for the CT‐based patient geometry prior to abovementioned steps, since the AXB algorithm performs explicit simulation of physical interactions in matter. Material type and mass density are defined for each voxel in the mapping, applying CT simulator specific CT number‐to‐mass density conversion curve and material assignment library provided with the algorithm. The material library includes five tissue types and 16 other materials, the mass density upper limit being 8.0 g/cc for stainless steel.^(6)^ Therefore, the report mode for the final dose distribution is referred to as dose‐to‐medium in medium ($D_{m,m}$). Even though the AXB algorithm inherently calculates $D_{m,m}$, the dose distributions can be converted to dose‐to‐water in medium ($D_{w,m}$), which is done by replacing the medium‐based fluence‐to‐dose response function used in absorbed dose calculation with a water‐based response function. In the pencil beam convolution (PBC) algorithm (type ‘a’ algorithm) and in the AAA (type ‘b’ algorithm), which are implemented in Eclipse TPS, the dose report mode is also $D_{w,m}$, but in those algorithms the dose results are based on electron density‐based corrections applied to dose kernels calculated in water.^6^, ^11^, ^23^ Therefore $D_{w,m}$ mode of the AXB algorithm represents more closely true absorbed dose‐to‐water.^(24)^

The main modifications between the AXB10 and the AXB11 are: 1) in the AXB11 air is included in the material library; 2) in the AXB11 the density ranges of the materials are slightly overlapping (Table 1); 3) in the AXB11 the image and contoured structure voxel alignment to calculation grid is improved; and 4) in the AXB11 the total (rest+kinetic) energy cutoff value for electron interactions is decreased from 1.011 MeV to 0.711 MeV. A more detailed and thorough description of the AXB algorithm is provided in the white paper from VMS by Failla et al.^(6)^ and in the first papers that feature the accuracy benchmarking of the original Acuros algorithm^(11)^ and the AXB algorithm.^12^, ^24^ Kan et al.^(25)^ have provided a review article on grid‐based LBTE solvers (i.e., the AXB algorithm), which presents the results of selected earlier papers describing the validation of the AXB algorithm for clinical use, and a comprehensive review article focusing on the accuracy of the AXB algorithm by Ojala^(5)^ covers all the published papers that incorporate the AXB algorithm.

**Table 1:** Mass density ranges for materials used in the AXB10,^(36)^ the AXB11,^(37)^ and MC calculations

	*MC model*	*AXB10*	*AXB11*
AIR521ICRU	0.0000‐0.0600	‐	0.0012‐0.0204
LUNG521ICRU	0.0600‐0.5900	0.0000‐0.5900	0.0110‐0.6242
ICRP adipose tissue	0.5900‐0.9850	0.5900‐0.9850	0.5539‐1.0010
ICRUTISSUE521ICRU	0.9850‐1.0750	0.9850‐1.0750	0.9693‐1.0931
ICRP cartilage tissue	1.0750‐1.4750	1.0750‐1.4750	1.0556‐1.6000
ICRPBONE521ICRU	1.4750‐2.2200	1.4750‐3.0000	1.1000‐3.0000
AL521ICRU	2.2200‐3.0000	2.2750−3.5600 ^a^	2.2750−3.5600 ^a^
Ti6Al4V	3.0000‐5.2400	3.5600−6.2100 ^a^	3.5600−6.2100 ^a^

a Manual material assignment.

### B. The MC model

The reference "full" MC simulations were performed with the BEAMnrc code package (V4–2.4.0, or BEAMnrc 2013), which uses the EGSnrc MC code system that simulates coupled electron–photon transport. The EGSnrc‐based phantom dose calculation is performed with DOSXYZnrc, which is also included in the BEAMnrc code package.^(26)^ The geometry model of the linac treatment head was based on the same Varian Clinac iX as in Materials and Methods section A. The MC model was based on the earlier work by the authors.^3^, ^4^, ^27^ The nominal photon beam energy for the MC model was 6 MV. The simulation of beam generation and transport in the linac treatment head was divided into two phases to allow the absolute dose calibration of the MC model, which was based on the concept by Popescu et al.^(28)^ The iterative initial electron beam tuning process and beam parameter selection are discussed in Ojala et al.^3^, ^27^

In the first phase of the simulation, the beam propagation through the static components of the treatment head was modeled, with the number of particle histories of $4\times 10^{7}$. The planar particle data information was collected into a phase space file, which was used as source for the second phase simulation through beam‐modifying components. This part was simulated as BEAMnrc shared library that was dynamically loaded by DOSXYZnrc code at run time. DOSXYZnrc source 20 in combination with synchronized beam‐modifying components as shared library allow the simulation of plans containing multiple fields or field segments (such as VMAT) in a single run.^(29)^ The plan parameters needed for MC simulation were exported from the TPS in DICOM‐RT format and converted to the form required by the MC code package. In the second phase and DOSXYZnrc simulations, the electron and photon transport cutoff parameters used were ECUT=AE=0.521 MeV and PCUT=AP=0.01 MeV. Other EGSnrc parameters were the same as in the earlier work by the authors.^(30)^ In each DOSXYZnrc simulation, the number of particle histories used, being from 1 to $5\times 10^{9}$, was selected so that the statistical uncertainty in high‐dose voxels was from 1.0% to 2.0%. The inherent dose report mode for the MC‐calculated dose distributions is $D_{m,m}$, which was also chosen for the AXB algorithm for better description of physical reality. However, for the readers interested in $D_{w,m}$ vs. $D_{m,m}$ differences of the AXB algorithm, the plans were also recalculated with dose report mode $D_{w,m}$ using the AXB11. The dose distributions were added to gamma analysis comparison presented in Results section. More thorough investigation on the $D_{w,m}$ vs. $D_{m,m}$ differences with the AXB algorithm has been performed by Rana and Pokharel.^(31)^

### C. Patient cases: CT datasets, material assignment and data analysis

For the patient CT dataset acquisition, Toshiba Aquilion LB 16‐slice model at 120 kVp (Toshiba Medical System, Otawara, Japan) was used, with varying imaging parameters depending on the patient anatomy. The scanner uses the 16‐bit depth for image pixels, yielding the extended CT scale. For the MC simulations, the CT‐based patient geometries were reconstructed with the CTCREATE code in DOSXYZnrc from sets of 1 to 3 mm thick CT slices, which were exported from the TPS. The CT number‐to‐mass density conversion curve, used both in the material assignment (Table 1) in the Eclipse TPS and in the MC calculations, was defined using the RMI Gammex 467 Tissue Characterization Phantom (Gammex, Middleton, WI) with additional aluminum and titanium‐aluminum‐vanadium alloy (Ti6Al4V) inserts with known atomic compositions and densities. Five different materials found in the default PEGS4 material library (AIR521ICRU, LUNG521ICRU, ICRUTISSUE521ICRU, ICRPBONE521ICRU, and AL521ICRU) and three additional materials (ICRP adipose tissue, ICRP cartilage tissue, and Ti6Al4V) created by the authors with PEGS4 utility found in the BEAMnrc code package, were assigned for the patient dataset voxels using the conversion curve. The corresponding cross‐section data for the materials were applied in MC dose calculation. All the above‐mentioned materials are implemented in the AXB algorithm material library and they are automatically assigned in the TPS calculations, excluding the manually assignable high‐Z structures with densities larger than $3.0\;g/{\text{cm}}^{3}$. The calculation grid sizes ranged between 0.1 to 0.2 cm, depending on the anatomical region, being always identical between the TPS and MC calculations, and fulfilling the recommendations for SBRT presented by Benedict et al.^(32)^

The plan data, including CT datasets, structure sets, and dose distributions calculated with the both AXB algorithm versions, were exported to CERR software, where also the MC‐recalculated dose distributions were imported. CERR, which uses MATLAB software (The MathWorks, Natick, MA) (version R2013a in this study), is a software package for the review and analysis of mainly radiotherapy planning data.^33^, ^34^ For the quantification of the discrepancies between the AXB10 and the AXB11 both algorithms were compared to the MC model through DVH analysis of the organs‐at‐risk (OARs) and target volumes (planning target volume (PTV) and gross/clinical tumor/target volumes (GTV/CTV)) applying clinically relevant criteria. Moreover, 3D gamma analysis tool within CERR software was applied between the MC model (reference dose) and the AXB algorithms (evaluation dose) for the 15% isodose region to allow overall plan comparison and for PTVs to quantify the differences in the volume to be treated. In 3D gamma analysis, following threshold criteria were set: 2% (of maximum dose of the reference dose) in dose difference and 1/1.5/2 mm in distance‐to‐agreement (DTA) (matching the DTA parameter value to the calculation grid size of each plan). To minimize the effect of inherent noise present in MC‐based dose distributions on gamma analysis results, large number of particle histories were simulated in MC calculations to minimize the statistical uncertainty and regions with less than 15% of maximum dose were neglected in the gamma analysis calculation, thus resulting in 15% isodose region. The results were presented with the gamma agreement index (GAI), which is the ratio of the number of calculation points passing the gamma test and the number of all calculation points. The patient cases with information on the PTV location and volume, fractionation scheme, treatment technique, and the calculation grid are shown in Table 2.

**Table 2:** Information on the selected patient cases

	*PTV Location*	*PTV volume (cc)*	*Fractionation*	*Treatment Technique*	*Grid (cm)*
Patient #1	Brain (2 mets)	20+5=25	5×6 Gy	VMAT 4 noncoplanar arcs	0.1
Patient #2	Brain	382	30×1.8 Gy	VMAT 2 arcs	0.1
Patient #3	Head & neck	320/1054	33×2/1.63 Gy	SIB‐IMRT 7 fields	0.15
Patient #4	Breast, left	1220	16×2.66 Gy	IMRT 2 fields	0.15
Patient #5	Lung, right, central	9	3×18 Gy	VMAT 7 arcs^a^, ^b^	0.15
Patient #6	Lung, left, lateral	52	8×7.5 Gy	IMRT 9 fields	0.15
Patient #7	Esophagus	203	28×1.8 Gy	VMAT 1 arc	0.15
Patient #8	Pancreas	149	5×6.6 Gy	VMAT 2 arcs	0.15
Patient #9	Vagina	1298	25×2 Gy	VMAT 2 arcs	0.2
Patient #10	Prostate (unilateral hip implant)	108	39×2 Gy	3 static conformal fields+VMAT (2 arcs)	0.2

a Flattening filter‐free (FFF) beams were not applied, since they were not available in the linac used in this study.

b Since in the linac used in this study there was a ‘MU per arc’ limitation, larger number of arcs than normally had to be used.

SIB‐IMRT = simultaneous integrated boost IMRT.

## III. RESULTS

The plans were divided in three groups depending on the anatomical location of the PTV. In the first group were the plans for Patients from #1 to #3. In Table 3 is shown the DVH analysis for the plans. For Patient #1 (brain, 2 mets) the MC simulations produced the lowest doses for PTV and GTV and slightly higher dose for the lens. The deviations with the AXB11 were smaller than with the AXB10, when compared to MC simulations. With Patient #2 (brain) the near maximum doses for PTV, GTV and OARs were larger with MC simulations, whereas mean and near minimum doses were close to each other with all calculation methods. For PTVs, GTV, and CTV of Patient #3 (head & neck), there were deviations between the MC simulations and AXB calculations, but in most of the cases the discrepancies were within 2%. With OARs the calculation produced similar results, except for the larynx, where the mean dose with MC simulations was about 3% lower than with the AXB10 and the AXB11.

In the second group were Patients from #4 to #7, for which the DVH data are shown in Table 4. With Patient #4 (breast) the mean dose to PTV was slightly higher with the MC simulations than with the AXB10 and the AXB11, but for the near maximum and minimum doses the AXB calculations were more than 2% lower than the MC simulations. For the OARs, all calculation methods produced nearly identical results. For Patient #5 (lung, central), which had the PTV of smallest volume in this study, the MC method predicted higher mean doses in PTV and GTV than the AXB10 and AXB11, the near maximum dose being over 2% higher for the GTV, while the doses to OARs were congruent between all the methods. The near minimum doses for the PTV and GTV of Patient #6 (lung, lateral) were more than 2% lower with the MC simulations than in the AXB10 and the AXB11 dose distributions, but for the mean values deviations were smaller, being almost negligible with the near maximum doses and for the OARs. With Patient #7 (esophagus), the AXB10 overestimated the dose level in the tracheal and esophageal air cavities, which led to the overestimation of the near minimum dose by almost 4%. However, the mean dose and near maximum dose for the PTV, and all the parameters for the GTV and OARs, showed negligible deviations with both AXB versions.

**Table 3:** DVH parameters for Patients from #1 to #3. The results are given as MC/AXB11/AXB10. The values are absolute doses (Gy). The values in bold for PTV/GTV/CTV differ more than 2.0%, when compared to the MC simulations

*Structure*	*Parameter*	*Patient #1*	*Patient #2*	*Patient #3*
PTV1 (PTV2)	$D_{95\%}$	29.7 / **30.3** / **30.7**	50.9 / 50.9 / 51.3	59.6(50.1) / 59.8(**48.9**) / **58.2**(49.3)
	$D_{\text{mean}}$	34.4 / 34.9 / **35.3**	53.4 / 53.0 / 53.2	65.2(54.4) / 65.2(54.4) / 64.9(54.6)
	$D_{2\%}$	38.2 / 38.7 / 38.9	56.1 / **54.4** / **54.6**	68.8(64.0) / 68.5(63.7) / 68.8(63.7)
GTV (CTV)	$D_{95\%}$	33.4 / **34.1** / **34.5**	52.1 / 52.7 / 53.0	58.9(52.1) / **60.2**(52.4) / 57.9(52.6)
	$D_{\text{mean}}$	35.6 / 36.2 / **36.6**	54.0 / 53.5 / 53.7	65.6(55.3) / 65.8(55.3) / 65.3(55.5)
	$D_{2\%}$	38.3 / 38.8 / 39.0	56.4 / **54.5** / **54.6**	68.9(64.3) / 68.9(63.7) / 69.3(63.8)
Lens	$D_{\text{mean}}$	0.57 / 0.46 / 0.46	3.3 / 3.4 / 3.3	‐
Medulla	$D_{0.1\text{cc}}$	‐	‐	45.6 / 45.8 / 45.8
Parotid gland	$D_{\text{mean}}$	‐	‐	26.3 / 26.5 / 26.6
Larynx	$D_{\text{mean}}$	‐	‐	45.8 / 47.0 / 47.2
Brainstem	$D_{0.1\text{cc}}$	‐	53.1 / 51.6 / 52.1	45.7 / 46.0 / 46.4
Optical chiasm	$D_{0.1\text{cc}}$	‐	34.4 / 31.3 / 32.3	‐
Optical nerve	$D_{0.1\text{cc}}$	‐	15.8 / 14.1 / 14.5	‐

**Table 4:** DVH parameters for Patients from #4 to #7. The results are given as MC/AXB11/AXB10. The values are absolute doses (Gy) (except with parameter $V_{20\text{Gy}}$). The values in bold for PTV/GTV differ more than 2.0%, when compared to the MC simulations

*Structure*	*Parameter*	*Patient #4*	*Patient #5*	*Patient #6*	*Patient #7*
PTV1	$D_{95\%}$	37.9 / **33.3** / **34.3**	54.3 / 53.5 / 54.6	51.8 / **53.2** / **53.1**	46.8 / **47.9** / **48.7**
	$D_{\text{mean}}$	41.9 / 41.2 / 41.3	60.0 / 59.1 / 60.0	67.1 / 67.4 / 67.8	50.4 / 50.1 / 50.6
	$D_{2\%}$	45.0 / **44.1** / **44.1**	66.3 / **64.7** / 65.5	74.8 / 74.6 / 74.9	53.5 / 53.0 / 53.3
GTV	$D_{95\%}$	‐	60.6 / 60.0 / 60.6	53.7 / **55.1** / **55.1**	48.9 / 48.6 / 48.9
	$D_{\text{mean}}$	‐	62.9 / 61.9 / 62.3	68.8 / 69.0 / 69.3	50.5 / 50.1 / 50.4
	$D_{2\%}$	‐	66.0 / **64.3** / **64.4**	75.0 / 74.7 / 75.0	53.6 / 52.9 / 53.2
Medulla	$D_{0.1\text{cc}}$	‐	7.0 / 6.9 / 7.0	4.8 / 4.7 / 4.8	33.9 / 33.5 / 33.5
Lung,^a^ Lung‐GTV^b^, Lungs‐GTV^c^	$D_{\text{mean}}$	7.4 / 7.4 / 7.4	3.9 / 3.8 / 3.9	10.0 / 9.9/ 9.9	9.1/ 8.9/ 9.0
	$V_{20\text{Gy}}(\%)$	15.4 / 15.7 / 15.6	4.3 / 4.2 / 4.4	17.5/ 17.3/ 17.3	7.9/ 7.8/ 7.9
Heart	$D_{\text{mean}}$	2.2 / 2.2 / 2.2	1.6 / 1.6 / 1.6	2.9 / 2.8 / 2.8	‐
	$D_{0.1\text{cc}}$	40.6 / 40.3 / 40.3	11.0 / 10.9 / 11.1	21.2 / 21.2 / 21.3	‐

a Values are for Patient #4.

b Values are for Patients #5 and #6.

c Values are for Patient #7.

In the third group were Patients from #8 to #10, for which the DVH data are shown in Table 5. For Patient #8 (pancreas), all the calculation methods produced consistent dose distributions for PTV, GTV, and OARs. With Patient #9 (vagina), the MC model produces higher overall dose level, most prominent in CTV and OARs, where deviations almost without exception exceed 2%. For Patient #10 (prostate with unilateral hip implant), the MC model predicts consistently about 0.5 to 1.4 Gy higher dose levels for PTV, CTV, and rectum, whereas for bladder all methods produce similar results.

**Figure 1:**
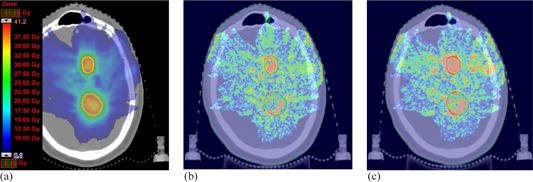
MC‐calculated dose distribution (a) and 3D gamma comparison map for MC vs. AXB11 (b) and MC vs. AXB10 (c) at the same plane for the Patient #1. In gamma maps red colour indicates voxels, where GAI exceeds 1.00 (i.e., test fails).

**Figure 2:**
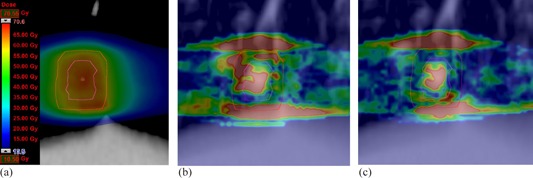
MC‐calculated dose distribution (a) and 3D gamma comparison map for MC vs. AXB11 (b) and MC vs. AXB10 (c) at the same plane for the Patient #5. In gamma maps red colour indicates voxels, where GAI exceeds 1.00 (i.e., test fails).

**Figure 3:**
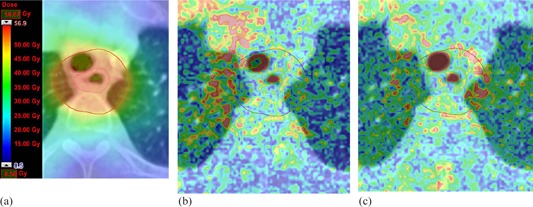
MC‐calculated dose distribution (a) and 3D gamma comparison map for MC vs. AXB11 (b) and MC vs. AXB10 (c) at the same plane for the Patient #7. In gamma maps red colour indicates voxels, where GAI exceeds 1.00 (i.e., test fails). The largest discrepancies are in the center of the air cavity (MC vs. AXB11/AXB10:5%/20%).

For all the patients, 3D GAI was evaluated for the dose distributions within the 15% isodose region and PTV. In general, both the AXB10 and the AXB11 produced GAI values close to 100% with all the plans, except with Patients #3, #4, and #9, for which the AXB11 gave equal or lower GAI values than the AXB10. For PTVs the variation in GAI values was much larger. The AXB11 resulted in larger or similar GAI values than the AXB10, except with Patients #2, #4, #5, and #9. Figure 1 (Patient #1) shows an example, where the GAI value for the AXB11 is larger than for the AXB10 in the PTV, whereas in Fig. 2 (Patient #5) the AXB10 resulted in larger GAI value than the AXB11 within the PTV. Figure 3 (Patient #7) shows a case, where the GAI values for the PTV do not differ much, but the large differences in dose distributions inside air cavity (trachea) result in larger deviations in DVH analysis for the PTV (Table 4). In DVH comparison (not presented) the dose distribution parameters calculated in dose report mode $D_{w,m}$ with the AXB11 were typically within 3%, without any clear trend, when compared to the dose report mode $D_{m,m}$. The overall discrepancies can be observed in the GAI values for the 15% isodose region. For the PTV, the GAI values were almost without an exception considerably lower with the dose report mode $D_{w,m}$ than with the $D_{m,m}$.

**Table 5:** DVH parameters for Patients from #8 to #10. The results are given as MC/AXB11/AXB10. The values in bold for PTV/GTV/CTV differ more than 2.0%, when compared to the MC simulations

*Structure*	*Parameter*	*Patient #8*	*Patient #9*	*Patient #10*
PTV1	$D_{95\%}\;(\text{Gy})$	31.3 / 31.1 / 31.4	48.7 / 47.8 / 48.0	76.4 / 75.2 / 75.2
	$D_{\text{mean}}\;(\text{Gy})$	34.8 / 34.8 / 34.9	51.0 / **50.0** / 50.1	79.1 / 78.2 / 78.0
	$D_{2\%}\;(\text{Gy})$	37.3 / 37.4 / 37.5	53.1 / 52.1 / 52.1	82.6 / 82.0 / 81.7
GTV^a^/CTV^b^	$D_{95\%}\;(\text{Gy})$	34.0 / 33.9 / 34.2	50.1 / **49.1** / 49.3	76.8 / 75.5 / 75.4
	$D_{\text{mean}}\;(\text{Gy})$	35.3 / 35.3 / 35.4	51.4 / **50.3** / **50.4**	79.0 / 78.2 / 77.9
	$D_{2\%}\;(\text{Gy})$	37.1 / 37.3 / 37.4	53.0 / **51.7** / **51.8**	82.1 / 81.6 / 81.3
Bladder	$V_{70\text{Gy}}(\%)$	‐	‐	16.2 / 16.1 / 16.1
	$V_{50\text{Gy}}(\%)$	‐	12.0 / 5.0 / 5.7	26.4 / 26.1 / 26.1
	$V_{30\text{Gy}}(\%)$	‐	59.4 / 57.9 / 58.4	‐
Rectum	$V_{70\text{Gy}}(\%)$	‐	‐	10.5 /9.3/9.5
	$V_{50\text{Gy}}(\%)$	‐	9.7 / 4.9 / 5.4	19.8 /18.3/18.6
	$V_{30\text{Gy}}(\%)$	‐	80.3 / 77.7 / 78.1	‐
Kidney	$D_{\text{mean}}\;(\text{Gy})$	4.4 / 4.2 / 4.3	‐	‐
	$V_{10\text{Gy}}(\%)$	13.2 / 12.3 / 12.8	‐	‐

a GTV values are for Patient #8.

b CTV values are for Patients #9 and #10.

## IV. DISCUSSION

When using the MC simulations as the reference, the results indicate that there are clinical situations, where both versions of the AXB algorithm produce dose distributions, with which the clinical acceptability may be compromised. This, of course, depends on the metrics used and the applied acceptance criteria. Since the AXB algorithm represents the most advanced class of dose calculation algorithms, a relatively stringent acceptance criterion of 2% for the DVH analysis and 2%/1−2 mm for 3D gamma analysis were chosen. In general, based on the results, no conclusion can be made as to whether the AXB11 would represent improved dose calculation accuracy over the AXB10, except within the air cavities. With Patients #1 (brain, 2 mets) and #7 (esophagus), the AXB11 outperformed the AXB10, which can be seen from DVH analysis (Table 3), especially in 3D gamma analysis for the PTV (Table 6) and also in Figs. 1 and 3, respectively. For Patients #3 (head & neck) and #6 (lung, lateral), the DVH analysis gave no clear preference one over the other when compared to the MC simulations, but in GAI values for the PTV, the AXB10 showed inferior congruence in comparison to the MC method. With Patients #4 (breast), #8 (pancreas), and #10 (prostate with unilateral hip implant), the DVH analysis and 3D gamma analysis showed no notable differences between the AXB10 and the AXB11 when compared to the MC simulations. For Patients #2 (brain), #5 (lung, central), and #9 (vagina), the AXB10 produced smaller deviations than the AXB11 in comparison to the MC method, which can be observed in the DVH analysis and most notably in the 3D gamma analysis for the PTV. For Patient #5 it is notable that, as shown by Fig. 2, the deviations occur in same regions than reported in previous work by the authors,^(3)^ and the AXB11 did not show better congruence with MC calculations, as expected. This was also observed for the Patient #6. Of all the plans, only for Patient #9 the GAI value of the 15% isodose region showed clear difference between the AXB10 and the AXB11, which was in favor of the former. It can be expected that the dose deviations are to some extent patient‐dependent. However, we consider that the results provided in this study give good impression of clinical usability of the investigated versions of the AXB algorithm.

**Table 6:** GAI values for dose distributions within the 15% isodose region contours and PTV using acceptance criteria 2%/1−2 mm (DTA parameter value equivalent to dose calculation grid resolution) for dose levels above 15% of the maximum dose in MC‐calculated dose distributions. The results are given in % as AXB11($D_{w,m}/\text{AXB}11/\text{AXB}10$

	*15% Isodose Region*	*PTV*
Patient #1	99.7 / 100.0 / 99.9	61.1 / 83.5 / 60.8
Patient #2	94.0 / 98.9 / 99.3	89.8 / 94.7 / 98.3
Patient #3	94.3 / 97.4 / 97.4	70.7 / 93.2 / 91.1
Patient #4	95.8 / 98.3 / 98.5	45.4 / 83.6 / 84.3
Patient #5	100.0 / 100.0 / 100.0	81.3 / 82.1 / 92.3
Patient #6	99.7 / 99.9 / 99.9	81.9 / 93.8 / 87.3
Patient #7	99.0 / 99.3 / 99.7	75.6 / 91.1 / 89.7
Patient #8	99.7 / 99.9 / 100.0	88.6 / 98.2 / 98.3
Patient #9	93.9 / 97.3 / 98.8	54.2 / 87.0 / 93.2
Patient #10	99.1 / 99.8 / 99.9	75.6 / 96.4 / 96.2

There are multiple explanations for the observed differences between the AXB calculations and the MC simulations and in the dose distributions between the two AXB algorithm versions. Firstly, there is a systematic discrepancy in the buildup dose calculation at the patient outer surface, which is observed as higher doses calculated by the MC method compared to AXB calculations. Presumably this is due to deficiencies in the multiple source model used by the AXB algorithm in electron contamination modeling and in different ways of how the calculation methods handle the alignment of the CT dataset, calculation grid, and the contoured structures. The effect of this issue can especially be observed as generally lower levels of GAI values for the 15% isodose region and PTV with Patients #3 and #4, where PTVs are in the proximity of the patient surface. Secondly, the same phenomenon as previously described is observable in air‐filled body cavities. As shown in Table 1, the AXB10 assigns air to very low‐density lung, which generates erroneous dose predictions, whereas air is defined as material in the AXB11. In this study it was observed that, with the AXB11, the dose levels are correctly predicted when comparing to the MC simulations. The largest deviations are in the edges of the air cavity, which is due to the slightly different material assignments (see Table 1) and the lower energy cutoff value for electron interactions with the MC model. This is shown in Fig. 3 for the Patient #7. This leads to general decrease in the GAI values for the 15% isodose region with Patient #3, for PTV with Patients #3 and #7, and for DVH analysis parameter $D_{95\%}$ for Patient #7. Thirdly, in regions, where the HU values fall to the lower end of the bone density range and to the higher end of the cartilage density range, discrepancies between the MC method and the AXB11 were observed. This is presumably due the modification in the material assignment of the AXB11 over the AXB10 that applies overlapping in the density ranges between two materials. The stopping power of the bone differs from other biological tissues insomuch that the $D_{m,m}$ difference compared to the MC method in these regions with mixed materials becomes observable. This decreases the general congruence between the AXB11 and the MC simulations and leads to lower GAI values for the 15% isodose region, especially with Patients #2, #3, #9, and for the PTV, especially with Patients #2, #3, #7, #9. For the dose distributions calculated with the dose report mode $D_{w,m}$, the deviations compared to the results of the $D_{m,m}$ are largest in the plans that contain large regions of assigned bone, adipose tissue, and other materials, which have stopping powers differing from water. The results on the $D_{w,m}$ vs. $D_{m,m}$ differences of this study support the findings by Rana and Pokharel.^(31)^

Previously described overlapping of density ranges between two materials in radiotherapy dose calculation algorithms is a new concept and, therefore, the question whether it represents better the reality, and thus more realistic dose distributions, is out of scope of this study. However, the traditional material assignment that is performed both with the MC simulations and also in the AXB10 (Table 1) may favor the results of the AXB10 over the results of the AXB11 in comparison to the MC method. Statistical noise is always present in MC‐based dose calculations and, if no smoothing techniques are applied, as in this study, in the dose distribution analysis the use of point doses must be avoided and noise may still add uncertainty to DVH parameters, such as near maximum and minimum values. In 3D gamma analysis, noise was seen as uniformly distributed sparse failed points of gamma test. The larger the dose calculation grid, the more observable this was, in spite of larger number particle histories simulated with plans containing larger PTVs (and thus larger treatment fields and larger calculation grids). This phenomenon was seen with Patients #3, #4, and #9 as lower GAI values, which has been also observed by Graves et al.^(35)^

With results of this work no explicit assessment can be made whether the modifications applied in the AXB11 over the AXB10 substantially improve the dose calculation accuracy. However, the inclusion of air in the material library showed expected improvements in the calculated dose distributions. The overlapping of the material density ranges is shown to have effects, but whether it represents more realistic dose prediction needs more detailed studies. The decreased energy cutoff value for electron interactions has more effect with lower material densities. This improved the GAI values for the PTVs with Patient #6, where the high‐density PTV is located in the low‐density lung, and with Patient #1, where the improved electron transport calculation in tumor/brain tissue interfaces has relatively larger effect with the small‐sized PTVs than in plans with larger PTVs. The improved GAI value was also expected with Patient #5 for the PTV in located centrally in the low‐density lung, but due to the small size and the relatively large movement of the tumor with respiration, despite of 4D CT applied, the HU values are blurred and, thus, the density of the GTV is lower, which decreases the effect of improved electron transport calculation.

## V. CONCLUSIONS

In this study, using full MC simulations as the reference, the effect of the modifications made in the AXB11 compared to the AXB10 on the dose calculation accuracy in the clinical patient treatment planning was assessed. However, no general conclusion can be made that the dose calculation accuracy of the AXB10 would be inferior to the AXB11, except in air cavities. The deviations between the two versions of the algorithm in the DVH analysis were generally small. Based on the results of 3D gamma analysis, no preference over the AXB11 or the AXB10 could be made. The effect of the improvements in the electron transport parameters and in material library applied in the AXB11 was only seen in dose distributions in air cavities, but otherwise it was perhaps covered by the effect of overlapping density ranges on the dose distributions. In addition, the separate density ranges, similar between the MC simulations and the AXB10, may favor the AXB10 in the comparisons and not reveal the potential calculation accuracy improvement with the AXB11. All in all, this study suggests that the results of the comprehensive studies assessing the accuracy of the AXB10 may be extended to the AXB11. Whether the extent of the discrepancies between the AXB and the MC simulations is to be decreased will be a question of modifications/improvements in future versions of the AXB and further similar studies.

## ACKNOWLEDGMENTS

The authors would like to thank Tony Popescu and Julio Lobo from British Columbia Cancer Agency, Vancouver Centre, for their assistance in extracting plan parameters, which were needed in MC simulations, from the Eclipse TPS. The authors declare that they have no conflicts of interest.
